# Rapid PCR-based method for herbivore dietary evaluation using plant-specific primers

**DOI:** 10.1371/journal.pone.0260105

**Published:** 2021-11-22

**Authors:** Arash Kheirodin, Mohammad Sayari, Jason M. Schmidt

**Affiliations:** 1 Department of Entomology, University of Georgia, Tifton, GA, United States of America; 2 Department of Plant Science, University of Manitoba, Winnipeg, MB, Canada; Natural Resources Canada, CANADA

## Abstract

Polyphagous pests cause significant economic loss worldwide through feeding damage on various cash crops. However, their diets in agricultural landscapes remain largely unexplored. Pest dietary evaluation in agricultural fields is a challenging task currently approached through visual observation of plant feeding and microscopic identification of semi-digested plant material in pest’s guts. While molecular gut content analysis using metabarcoding approaches using universal primers (e.g., rbcl and trnL) have been successful in evaluating polyphagous pest diet, this method is relatively costly and time-consuming. Hence, there is a need for a rapid, specific, sensitive, and cost-effective method to screen for crops in the gut of pests. This is the first study to develop plant-specific primers that target various regions of their genomes, designed using a whole plant genome sequence. We selected *Verticillium* wilt disease resistance protein (VE-1) and pathogenesis related protein-coding genes 1–5 (PR-1-5) as our targets and designed species-specific primers for 14 important crops in the agroecosystems. Using amplicon sizes ranging from 115 to 407 bp, we developed two multiplex primer mixes that can separate nine and five plant species per PCR reaction, respectively. These two designed primer mixes provide a rapid, sensitive and specific route for polyphagous pest dietary evaluation in agroecosystems. This work will enable future research to rapidly expand our knowledge on the diet preference and range of crops that pests consume in various agroecosystems, which will help in the redesign and development of new crop rotation regimes to minimize polyphagous pest pressure and damage on crops.

## 1 Introduction

Polyphagous mammalian pests [1] and insect pests [2] challenge the sustainability of agricultural production through direct crop damage and disease transmission [2]. Crop losses of up to 50–60% due to insect damage are reported from various parts of the world [2]. Therefore, it is of major importance to exercise all viable management options to minimize the impact of the polyphagous pest on agricultural production, which includes biological control, cultural control and habitat management strategies. Molecular gut content analysis (MGCA) is a powerful tool that broadens our understanding of trophic linkages among organisms in agroecosystems [3], which can advance these management strategies. To date, MGCA has been mostly employed to determine the diets of generalist predators [3–5], estimate the levels of insect parasitism [6–8], investigate intraguild predation [9, 10], with an aim to find important natural enemies of insect pests in agroecosystems. To a lesser extent, MGCA has been employed to determine the diet of insect herbivores in agroecosystems [11]. In this study, MGCA is employed to evaluate herbivore diet composition in agricultural fields. Given the significant contribution of various natural enemies to pest control services in the agroecosystems, these biological control-focused efforts are justified. However, more recently, an increasing body of evidence suggests crop diversity in agroecosystems can directly contribute to pest population control [12–14], regardless of natural enemies. Therefore, determining the range of crops consumed by particular pest species can help achieve a better understanding of their dispersal patterns [15], and develop more efficient crop rotation regimes and habitat management strategies to minimize pest damage to cash crops in agroecosystems [16, 17]. Such dietary information is vital for subtropical regions since unlike areas experiencing strong seasonality or those where only a few crops are grown, subtropical agricultural landscapes contain crops that are frequently rotated throughout the year [18]. Therefore, crop rotation and the right kind of crop diversity could play an important role in polyphagous pest population regulation in subtropical regions such as Southeast USA. Altogether, polyphagous pest dietary information could be used to add the right kind of crop diversity and manipulate crop planting dates to reduce pest pressure and damage to crops in agricultural landscapes.

Several approaches have been used to explore the dietary composition of insect pests, including visual observations of pest presence and plant damage in the field, microscopic gut content evaluation, and laboratory feeding trials [19]. Laboratory feeding trials may represent unrealistic host preferences because captive insect pests may only feed on the given plant species under laboratory conditions and not natural conditions [20]. Further, understanding the diet composition of polyphagous pests in agroecosystems is a challenging task using direct observation of feeding in agricultural fields, due to the time (e.g., nocturnal) and location of pest activity (e.g., under soil feeding) [19]. At the landscape scale, several techniques including bi-directional malaise traps [21], molecular mark-recapture [22–25] and isotope techniques [25–28] have been used to monitor the movement of polyphagous pests and their natural enemies between habitats, to understand how habitat preference or habitat composition shapes pest and natural enemy movement. However, while these techniques could provide useful information regarding the movement of pests and natural enemies among crop and habitat patches in the landscape, it does not reveal the diet breadth of polyphagous pests and natural enemies, or their intention for visiting different crop patches in the landscape (e.g., to feed on insect prey or plant carbohydrate resources). Employing MGCA, however, can increase the level of accuracy of identifying ingested plant DNA in herbivores. Thus, building diagnostic MGCA for identifying plant DNA in herbivores will help resolve host- polyphagous pest interactions.

Developing PCR-based molecular gut content analysis (MGCA) is now a cost-effective strategy to study complex ecological interactions (e.g. predator-prey, herbivore-plan, pollinator-host interactions) in agroecosystems [3, 4, 29, 30]. MGCA allows researchers to obtain dietary information of pests and their natural enemies by tracking the insect and plant DNA detection frequency in natural contexts. Several techniques employ MGCA to determine polyphagous pest diets in agricultural fields, including restriction fragment length polymorphism (RFLP) [31, 32], singleplex [33] and multiplex PCR [34–37], and metabarcoding approaches [20, 38–41]. MGCA of herbivorous insects using metabarcoding with universal plant primers (e.g., rbcl and trnL) has allowed comprehensive analysis of polyphagous pest gut content to examine host plant diversity and preference [20, 38–41]. While metabarcoding is very sensitive to small degraded quantities of digested plant DNA in the polyphagous pest’s gut [19], it comes with the following dependency on bioinformatics expertise, and relatively high sequencing costs [29]. Similarly, RFLP allows the separation of several species in a sample using restriction enzymes [31, 32]. However, the technique becomes less sensitive when a high number of species are present in samples due to the presence of similar amplicon sizes within the gut content, which results in overlapping bands and detections [29]. Multiplex PCR is a powerful tool that allows rapid and precise identification of several target species in one PCR reaction. The major difficulty of the technique is designing primer mixes that are sensitive (e.g., detecting a low concentration of target DNA in the mix), specific (e.g., amplify only the target and no non-target species in the mix) and compatible with one another within the mix. However, once optimized, multiplex PCR offers a rapid and cost-effective solution for estimating dietary preferences of polyphagous pests and predators.

The plant chloroplast genome sequence is typically used for developing specific and universal plant primers for polyphagous pest dietary evaluations and plant phylogenetic analysis [29, 42–44]. However, due to low variability in the chloroplast genome sequences among the target plant species, we used a whole plant genome approach on multiple genes to develop and optimize a multiplex PCR approach for documenting plant feeding by polyphagous pests. Currently, MGCA protocols for plants are rare for crop plant detection in polyphagous pest’s gut (however, see [24, 33]). While several non-crop species-specific plant primers were previously reported [45], to our knowledge, there is only one study detailing a multiplex PCR approach for crop and non-crop plant detection in the gut contents of pests [29]. Following the development of the multiplex PCR primer mixes, the same group has successfully documented herbivorous feeding frequencies of wireworms in wheat and veggie landscapes [34–37]. Using molecular dietary evaluation, Staudacher et al. [34] found a significant decrease in maize feeding and damage by wireworms in diversified cropping systems with grass and legume relative to maize monoculture, which translated to higher maize yield. This result suggests that crop diversification could result in lower pest pressure and damage to field crops, supporting the resource concentration hypothesis [14] and demonstrating how MGCA with multiplex primer sets could answer the important ecological and applied question in agricultural fields.

While these initial contributions to understanding polyphagous pest feeding are encouraging, the targeted plants are not representative of several important cash crops in agroecosystems. Here we present the first method using whole plant genome sequences to design specific primers for 14 important crop species in the world and the USA [18], that enables polyphagous pests (e.g., animal pests, birds and insect pests) dietary determination in agroecosystems. Such information can be used in association with the levels of crop and habitat diversity in the surrounding landscapes to find the best crop rotation and habitat management strategy to reduce pest damage to cash crops. Our objectives in this study were to 1) design specific and sensitive plant primers for fourteen important crop species worldwide, 2) optimize and create efficient, low-cost multiplex PCR primer mixes that are compatible, specific and sensitive towards their target plant species, 3) determine the plant DNA detectability half-life in *M*. *ponderosus* gut through environmentally controlled laboratory feeding trials, and 4) screen field-collected *M*. *ponderosus* individuals using the optimized primer mixes to determine the polyphagous pest’s diet under field conditions.

## 2 Materials and methods

### 2.1 Case study

This study is part of a comprehensive project to unravel polyphagous pest diets (e.g., birds and insect pests) in the Southeast USA, and part of a large-scale landscape study to understand polyphagous pest movement across (or between) habitats within agricultural landscapes.

Given the high abundance and damage of grasshoppers to agricultural fields [46] and due to the fact that grasshoppers are considered polyphagous pests that feed on various crops [47], we used the spur-throat grasshoppers, *Melanoplus ponderosus*, as a model to test and evaluate the efficacy and specificity of the designed primers. *M*. *ponderosus*, was targeted to understand polyphagous pest feeding in agricultural mosaics containing cropland cover where pests may move between crops. This species was selected due to 1) its high abundance in agricultural fields in our study area, 2) the generalist diet status of grasshoppers that are known to feed on several crops and non-crops [47], and 3) worldwide there is a demand to understand grasshopper diets and food webs in agricultural and native lands.

### 2.2 Primer design

The most common annual crops with the highest acreage in the Southeast USA were selected for primer design (Table 1) [18]. The plant chloroplast genome sequence is commonly used for phylogenetic analysis and species identification of plants [42–44]. In this study, we first tried to use available regions from chloroplast genomes in common crops using two well-known genes (*ie*; trn-L, trn-F). However, due to the high conservation of chloroplast genomes compared to nuclear and mitochondrial genomes, and due to difficulty in finding a specific region in conserved parts of the genome in different plants that we used in this research, we were not able to find a region that could be used to design plant-specific primers. We searched the full genome sequences to find a region to design plant-specific primers to overcome this issue. For this purpose, we used the full genome sequences of 12 different plants, including *Solanum lycopersicum*, *Gossypium hirsutum*, *Phaseolus vulgaris*, *Citrullus lanatus*, *Cucumis sativus*, *Cucurbita pepo*, *Solanum melongena*, *Glycine max*, *Zea mays*, *Solanum tuberosum*, *Sorghum bicolor* and *Arachis hypogaea* (Table 1). Full genome alignments showed suitable regions to design plant-specific primers. Based on our analysis, *Verticillium* wilt disease resistance protein (VE-1) genes for *S*. *lycopersicum*, pathogenesis related protein-coding gene 2 (PR-2) in squash and PR-4 in all other species were chosen as good targets for plant-specific primer design. These regions allowed us to easily find species specific regions as we could see high variability in the sequences amongst different plant species used in this research. Since there are no available genomes for onion (*Allium cepa*) and sweet potato (*Ipomoea batatas*), we used the full sequences of onion as well as sweet potato’s PR-5, PR-1 and PR-2 in all our alignments to find the best fragment for these plants. Whole genome sequence alignments were performed using MAFFT (Multiple Alignment using Fast Fourier Transform; [48]) using the default parameters. After choosing proper regions based on the genome alignments, we used BioEdit [49] to hand-edit the gene alignments. From the alignments, we found regions specific to each of the 14 species and could be used as a target for specific primer design. For primer design, we used CLC Genomic Workbench version 11.0.1 (Qiagen Bioinformatics, Aarhus, Denmark). Finally, we did a blast search of each primer against all other plant genomes to ensure specificity on target plants. In summary, we designed 14 pairs of species-specific primers by leveraging the available genomes of 12 plant species and gene sequences from two additional species (Table 2).

**Table 1:** Isolate numbers and genome sequence information used for species specific multiplex primer design.

Species	GenBank Accession number	References
***Solanum lycopersicum***	AEKE00000000	[50]
***Gossypium hirsutum***	LBLM00000000	[51]
***Phaseolus vulgaris***	ANNZ00000000	[52]
***Citrullus lanatus***	VOOL00000000	[53]
***Cucumis sativus***	ACHR00000000	[54]
***Cucurbita pepo***	NHTM00000000	[55]
***Solanum melongena ***	BAUE00000000	[56]
***Glycine max***	ACUP00000000	[57]
***Zea mays ***	CABHLF000000000	[58]
***Solanum tuberosum***	AEWC00000000	[59]
***Sorghum bicolor***	ABXC00000000	[60]
***Arachis hypogaea***	PIVG00000000	[61]

**Table 2:** List of designed primers along with detailed information regarding their target plant species, primer sequences, expected band sizes, optimized annealing temperature (°C), and their concentration (μM) within multiplex primer mixes.

Target plant species: common names	Primer names	Primer sequences (5’-3’)	Plant genome region	Product size (bp)	Annealing temperature	Primer conc in mix (μM)	Multiplex mixes
*Cucurbita pepo*	CuPE-FP	GCCAAAGATTGCCAGATGGTC	PR-2 genes	115	59.86	0.2	Mix 1
Squash	CuPE-RP	CCCACATTTGAACTGCGTCA			59.05		
*Allium cepa*	AlCE-FP	TTGAAAACCGCTACTGGCCT	PR-5 genes	145	59.89	0.3	Mix 1
Onion	AlCE-RP	AATATACTGGGGCCGGGGA			59.76		
*Glycine max*	GlMA-FP	ATGCCACCAAGGCCAAGAC	PR-4 genes	200	60.61	0.2	Mix 1
Soybean	GlMA-RP	AGGGACAACCGTGTTAGCATA			59.10		
*Ipomoea batatas*	IpBA-FP	TTGGTTTACACGACCCGGTG	PR-1 genes	220	60.53	0.3	Mix 1
Sweet potato	IpBA-RF	TGCACCGACAAATAACAGCG			59.48		
*Phaseolus vulgaris*	PhVU-FP	AACACCACAGAGAGTGTTGGG	PR-4 genes	250	60.13	0.3	Mix 1
Bean	PhVU-RP	CAGCTTCGCAATACAGGTGC			59.90		
*Gossypium arboretum*	GoAR-FP	GGGCACTTCAAAGGAAAGCAG	PR-4 genes	270	60.00	0.2	Mix 1
Cotton	GoAR-RP	TCCAGTGTCGCAAACCACTC			60.53		
*Citrullus lanatus*	CiLA-FP	CTACTGGGCAAATTCTTGCGT	PR-4 genes	330	59.19	0.2	Mix 1
Watermelon	CiLA-RP	GTGAAGTATGACAAAGACATGAACA			57.81		
*Solanum lycopersicum*	Soll-FP	TGCACACAAACACAAGATAGAGG	VE-1 genes	380	59.19	0.2	Mix 1
Tomato	Soll-RP	TGCGAGGAAAGTCCAAAACAC			59.33		
*Arachis hypogaea*	ArHY-FP	GCTTACTCTCAAGTACCACACCA	PR-4 genes	405	59.99	0.2	Mix 1
Peanut	ArHY-RP	AGCTGCAGCAGATAGAAGGC			60.18		
*Zea mays*	ZeMA-FP	GGCGAGAGCCCCTACTAGA	PR-4 genes	180	59.85	0.4	Mix 2
Corn	ZeMA-RP	CACAAATCGCCTGCATGGTT			59.76		
*Solanum melongena*	SoME-FP	TGACTGGGTGCTTTGTCGAA	PR-4 genes	325	59.82	0.3	Mix 2
Eggplant	SoME-RP	CATGAGTCGGAACCTGAGCC			60.46		
*Solanum tuberosum*	SoTU-FP	ATGTCTTGGGATGCCGGTTT	PR-4 genes	340	59.46	0.4	Mix 2
Potato	SoTU-RP	AGTAAGGACGTTGTCCGACC			59.40		
*Sorghum bicolor*	SoBI-FP	GACATGCGGTACCAGTTCCT	PR-4 genes	350	59.82	0.4	Mix 2
Sorghum	SoBI-RP	CTGCCATTGTAGCATGTGACC			59.90		
*Cucumis sativus*	CuSA-FP	CCCCATTCTCCTCCTCCTAAC	PR-4 genes	407	58.95	0.3	Mix 2
Cucumber	CuSA-RP	TCTAGCACATCCAATCCGGC			59.89		

### 2.3 Plant preparation and DNA extraction

Fresh plant material was collected from agricultural fields and kept in a -20 freezer until extraction. Before extraction, 100 mg of plant leaf tissue was washed using 10% bleach, molecular grade H2O, and 95% ethanol to ensure no cross-plant DNA contamination was present on the leaves. Samples were frozen using liquid nitrogen and then ground to a fine powder using sterilized ceramic mortar and pestles. Then, DNA was extracted following manufacturer protocols for Qiagen DNeasy Plant Mini Kit (QIAGEN, Chatsworth, CA, USA).

### 2.4 Primer specificity and sensitivity testing

For in silico primer specificity testing, all primer sets were blasted against the NCBI database using Primer-BLAST with the following settings [62]: targeting organisms matching the class of plants, Insecta and spiders. We limited our search to a maximum of one mismatch to the target species and up to four mismatches to the non-target species and ignored any hit with more than four mismatches to the blasted primer sets.

Furthermore, for each primer pair, we performed in vivo specificity test in the laboratory to test the potential cross-amplification on 15 non-target plant and insect species (Table 3). The non-target plant species (e.g., plants that we did not design specific primers for) were selected based on their availability in Southeast production landscapes, which included several crops and non-crop plants (Table 3). Plants were tested using the 14 primer pairs against all non-target species to ensure that the designed primers did not amplify non-target plant DNA. In brief, each primer pair was initially tested on the target plant and then tested against the combined DNA from all non-target plants (non-target plants included and not included in our multiplex primer mixes, (Table 3)). The specificity of the primer to amplify its target DNA fragment in the multiplex primer mix was evaluated by testing the primer mix on their mixed intended target DNA (9 and 5 plant species DNA for mix 1 and 2 respectively), and target DNA within the mix of 15 non-target plants DNA’s (Table 3). This was done to ensure that the primer set was able to amplify its target DNA both when only the target DNA was present and when pooled with non-target DNA samples. The sensitivity of each primer set was further evaluated by diluting the extracted plant DNA into four ten-fold serial dilutions (1:10–1:10000). All primer set sensitivity tests were done in triplicate. The primer sensitivity test was performed on individual primer sets as well as mixed primer sets to ensure primer sensitivity within the primer mix. The concentration of DNA in the diluted samples was estimated using a Spectra Max Gemini XPS microplate reader (Molecular Devices, LLC, San Jose, California, USA).

**Table 3:** List of target and non-target plant and insect species used to test for individual primer specificity evaluations using singleplex PCR.

Target tested	Non-target tested
**Plant species tested**	**Plant species tested**
*Solanum lycopersicum;* Tomato	*Vigna unguiculate;* Black-eye peas
*Gossypium hirsutum;* Cotton	*Vigna unguiculate;* Blue lake snap peas
*Phaseolus vulgaris;* Beans	*Abelmoschus esculentus;* Okra
*Allium cepa;* Onion	*Vigna unguiculate*; Zipper peas; Lady Finger peas
*Glycine max;* Soybean	*Phaseolus lunatus*; Butter beans
*Citrullus lanatus;* Watermelon	*Capsicum annum*; Jalapenos pepper; Bell pepper
*Cucurbita pepo*, Squash	*Cucumis melo;* Athena cantaloupe
*Arachis hypogaea;* Peanut	*Rudbeckia hirta*; Susan
*Ipomoea batatas;* Sweet potato	*Prunus persica*; Peach
*Solanum melongena;* Eggplant	*Clitoria ternatea*, Butter peas
*Sorghum bicolor; Sorghum*	*Brassica oleracea*; Collard, cabbage, broccoli
*Cucumis sativus;* Cucumber	*Brassica rapa*; Mustard, canola, oilseed
*Solanum tuberosum;* Potato	**Insect species tested**
*Zea mays;* Maze, corn	*Bemisia tabaci*; Whitefly adult
	Acrosternum hilare, Stink bug adult
	*Melanoplus ponderosus*, Grasshopper

**Note:** None of the non-target species tested were amplified by any of the primers under singleplex or multiplex tests of cross-reactivity

### 2.5 PCR optimization and multiplex primer mix testing

The PCR reactions were carried out in a Bio-Rad C1000 Touch © Thermal Cycler (Bio-Rad, Hercules, California USA). Initial PCR’s were performed using four different volumes of target DNA and PCR grade water: 1, 2, 2.5 and 3.5 μL of target DNA and 3.65, 2.65, 2.15 and 1.15 μL of PCR grade water, respectively. The best quality of bands and detection was achieved with 3.5 μL of target DNA. Hence, the PCR components were optimized as follows: 6.25 μL of 2x Qiagen Multiplex Master Mix (Qiagen, Hilden, Germany), 0.1 μL of 5xQ-solution (Qiagen, Hilden, Germany), 0.25 μL of Bovine Serum Albumin (Thermo Scientific, Waltham, MA USA), 1.15 μL of PCR grade water, 1.25 μL of primer mix, and 3.5 μL of DNA, to the total volume of 12.5 μL. The PCR reaction cycle started with an initial denaturation at 95°C for 15 min, followed by 34 cycles of 30 s denaturation at 95°C, 90 s annealing at 60.0°C, 90 s of extension at 72°C and a final extension at 72°C for up to 10 min. The PCR products were visualized using QIAxcel Advanced Systems (Qiagen®).

Primer mixes were created and tested based on the primers target product size, compatibility, sensitivity and specificity towards their target DNA. Step-wise testing was performed to adjust the concentration of primers within each mix to ensure standardized sensitivity of all primer pairs for their target plant DNA.

### 2.6 Half-life detectability

#### 2.6.1 Sample collection

For field sites, we used land associated with the College of Agriculture & Environmental Sciences (CAES) Research and Education Centers (RECs), University of Georgia, Tifton, Georgia (31°32’05” N 83°24’24” W). The CAES mission for the RECs is to provide experimental land for all UGA scientists and students to enhance knowledge and education of agriculture and outreach to the public. *M*. *ponderosus* individuals were collected using sweep net sampling from grassy areas, and were immediately transferred to sterile cylinder shape containers. The containers contained two square-shaped holes on the sides and transparent lead covered by mesh, to allow airflow. In the laboratory, the individual grasshoppers were provided with wet filter paper to provide humidity and stored in growth chambers (25°C, L16: D8), and starved for 48 hours before the feeding trial. The filter papers were changed every day to maintain the humidity in the containers.

#### 2.6.2 Experimental setting

The experiment was conducted under controlled conditions (25°C, L16: D8). As a food source, cotton leaves were collected from the University of Georgia experimental plots at the Tifton campus within cotton fields receiving no insecticide treatments. To standardize the amount of plant material, each grasshopper was provided with a rectangular piece of cotton leaf (3 cm length * 1 cm width) and kept under continuous observation until the entire leaf was consumed. Prior to the trial, each container was randomly assigned to a time interval. All individuals were kept under continuous observation, and the exact time at which the individual consumed the leaf fragment was recorded. Seven-time intervals were tested, including: 0, 12, 24, 36, 48, 72 and 96 hours after feeding (n = 10 per time interval). Following observed consumption at each time interval, individuals were immediately transferred to sterile vials containing prechilled 95% ethanol and stored at -20° C until DNA extraction.

### 2.7 Grasshopper diets in Georgia agroecosystems

Ninety-four individual *M*. *ponderosus* were collected by sweep-net at six locations within the CAES REC near Tifton, GA. Individuals were collected from grasslands located between various agricultural crop fields (e.g., corn, cotton, peanut fields) to increase the chance of mix plant DNA diet in grasshopper guts. Immediately after collection, each individual was transferred to individual 20 ml disposable scintillation vials (Fisher®) containing 95% ethanol and placed inside a cooler box to avoid DNA contamination and degradation. In the laboratory, the vials were kept at -20° C and stored until extraction.

### 2.8 Grasshopper preparation and DNA extraction protocol

Frozen grasshoppers from the laboratory feeding trials and the field collections were transferred to sterile Petri-dishes (60 × 15 mm, Falcon®) and air-dried for ~30 seconds. The specimens were then dissected using (sterile) surgical scissors and forceps, and the entire digestive parts (e.g., crop and colon) were transferred to individual Eppendorf 1.5 mL vials. After each dissection, the scissor and forceps were carefully washed with 10% bleach, molecular grade water, 95% ethanol and flame sterilized. The DNA was extracted from dissections using Qiagen DNA blood and tissue extraction kits (Qiagen, Hilden, Germany) according to the manufacturers protocol with slight modifications. In the final DNA extraction steps, instead of an overall 200 μL of room temperature buffer AE, 150 μL of ~62 C° AE buffer was used in an attempt to extract a higher concentration of the plant DNA. Each sample was then processed using the optimized multiplex PCR protocols to screen for plant DNA.

### 2.9 Statistical analysis

We conducted all the statistical analyses in R version 3.6.3 [63]. The detectability of cotton DNA in the gut of *M*. *ponderosus* was calculated using probit regression. Probit analysis allows identifying the time period at which DNA is traceable in the gut content of consumers [36]. The binary presence/absence data of cotton DNA in the *M*. *ponderosus* gut was the dependent variable and time intervals after leaf fragment consumption was the independent variable. We fit the binomial presence/absence cotton DNA data over six-time intervals to a probit regression model to predict the half-life (i.e. the time at which the cotton DNA is traceable with 50% probability) for cotton DNA in grasshopper’s gut.

The MGCA result from the field-collected grasshopper was used to estimate the percent of grasshopper positives for each plant species and the total percentage positives for plant DNA. The percentage of *M*. *ponderosus* positives for each target plant species was estimated by dividing the number of positives for each target plant by the total number of individuals tested. Total percentage of positives for plant DNA was calculated by dividing the number of individuals positive for all plant target species by the total number of the individuals tested.

## 3 Results

### 3.1 Primer specificity and sensitivity

The in-silico primer specificity testing in NCBI Primer-Blast indicated high specificity of the designed primers to their target plants, but no non-target plant, insect or spider species. The only exception was *Solanum tuberosum* that could also hit *Capsicum annuum* with 3 mismatches. All other primers only targeted their intended plant species sequences with up to 99% similarities and no other species. In vivo primer testing confirmed this, as none of the designed primers amplified any of the 28 non-targets (e.g., 13 plants that we designed primer for in this study along with 15 additional tested plants and insects; Tables 2 and 3). All primer pairs were designed to have annealing temperatures around 60°C, and as expected, this annealing temperature yielded the best quality bands. Further, in-vivo specificity tests indicated that the multiplex primer mixes did not amplify any of the 15 non-target plant and insect species, indicating high specificity within the mix of primers. The primer sensitivity test further showed that all designed primers were capable of amplifying their target plant DNA in concentrations as low as 0.219 plant DNA ng/μL. The majority of primers were capable of amplifying target plant DNA in concentrations as low as 0.144 plant DNA ng/μL (Table 4). Corn, sorghum and bean were among the least sensitive primer pairs when tested in the mixes, and their concentration in two primer mixes was adjusted to standardized the mix. By adjusting each primer pair concentration based on the sensitivity of each primer within the mix, we standardized the mix to enhance the efficacy of each primer (Table 2). Our results further indicate that all primers within these two mixes are compatible, and can detect target DNA without masking the performance of other primers within the mixes.

**Table 4:** The result of in-silico NCBI Primer-BLAST, indicating the number of matches and number of mismatches with the intended target and non-target hits, along with sensitivity of the specific primers both individually and when used in the mix estimated by Spectra Max Gemini XPS microplate reader.

Target plant species: common names	Primer names	Hits to plants	Hits to Insecta	Sensitivity single primer	Sensitivity in primer mix
*Solanum lycopersicum*	Soll-FP	*Solanum pennellii*, wild tomato, one mismatch, *S*. *lycopersicum*, no mismatch	None	0.140 DNA ng/μL	0.140 DNA ng/μL
Tomato	Soll-RP				
*Gossypium arboretum*	GoAR-FP	*G*. *arboretum*, *G*. *hirsutum* and *G*. *raimondii *with no mismatch	None	0.125 DNA ng/μL	0.125 DNA ng/μL
Cotton	GoAR-RP				
*Phaseolus vulgaris*	PhVU-FP	*Phaseolus vulgaris* with no mismatch	None	0.125 DNA ng/μL	0.185 DNA ng/μL
Bean	PhVU-RP				
*Ipomoea batatas*	IpBA-FP	None. The genome is not available in NCBI database	None	0.161 DNA ng/μL	0.122 DNA ng/μL
Sweet potato	IpBA-RF				
*Allium cepa*	AlCE-FP	None. The genome is not available in NCBI database	None	0.122 DNA ng/μL	0.122 DNA ng/μL
Onion	AlCE-RP				
*Glycine max*	GlMA-FP	*Glycine max* with no mismatch	None	0.144 DNA ng/μL	0.144 DNA ng/μL
Soybean	GlMA-RP				
*Citrullus lanatus*	CiLA-FP	The genome used for primer design is not available in NCBI. None	None	0.122 DNA ng/μL	0.113 DNA ng/μL
Watermelon	CiLA-RP				
*Cucurbita pepo*	CuPE-FP	*C*. *moschata* with one mismatch, and *C*. *pepo* with no mismatch	None	0.219 DNA ng/μL	0.113 DNA ng/μL
Squash	CuPE-RP				
*Arachis hypogaea*	ArHY-FP	*A*. *hypogaea* and *A*. *ipaensis* with no mismatch	None	0.131 DNA ng/μL	0.131 DNA ng/μL
Peanut	ArHY-RP				
*Solanum melongena*	SoME-FP	The genome used for primer design is not available in NCBI. None	None	0.180 DNA ng/μL	0.180 DNA ng/μL
Eggplant	SoME-RP				
*Zea mays*	ZeMA-FP	*Zea mays* with no mismatch	None	0.203 DNA ng/μL	0.203 DNA ng/μL
Corn	ZeMA-RP				
*Solanum tuberosum*	SoTU-FP	*Capsicum annuum* with 3 mismatch, and *S*. *tuberosum* with no mismatch	None	0.151 DNA ng/μL	0.151 DNA ng/μL
Potato	SoTU-RP				
*Sorghum bicolor*	SoBI-FP	*Sorghum bicolor* with no mismatch	None	0.210 DNA ng/μL	0.210 DNA ng/μL
Sorghum	SoBI-RP				
*Cucumis sativus*	CuSA-FP	Cucumis sativus with no mismatch	None	0.172 DNA ng/μL	0.219 DNA ng/μL
Cucumber	CuSA-RP				

### 3.2 Multiplex primer mixes optimization

**Fig 1:**
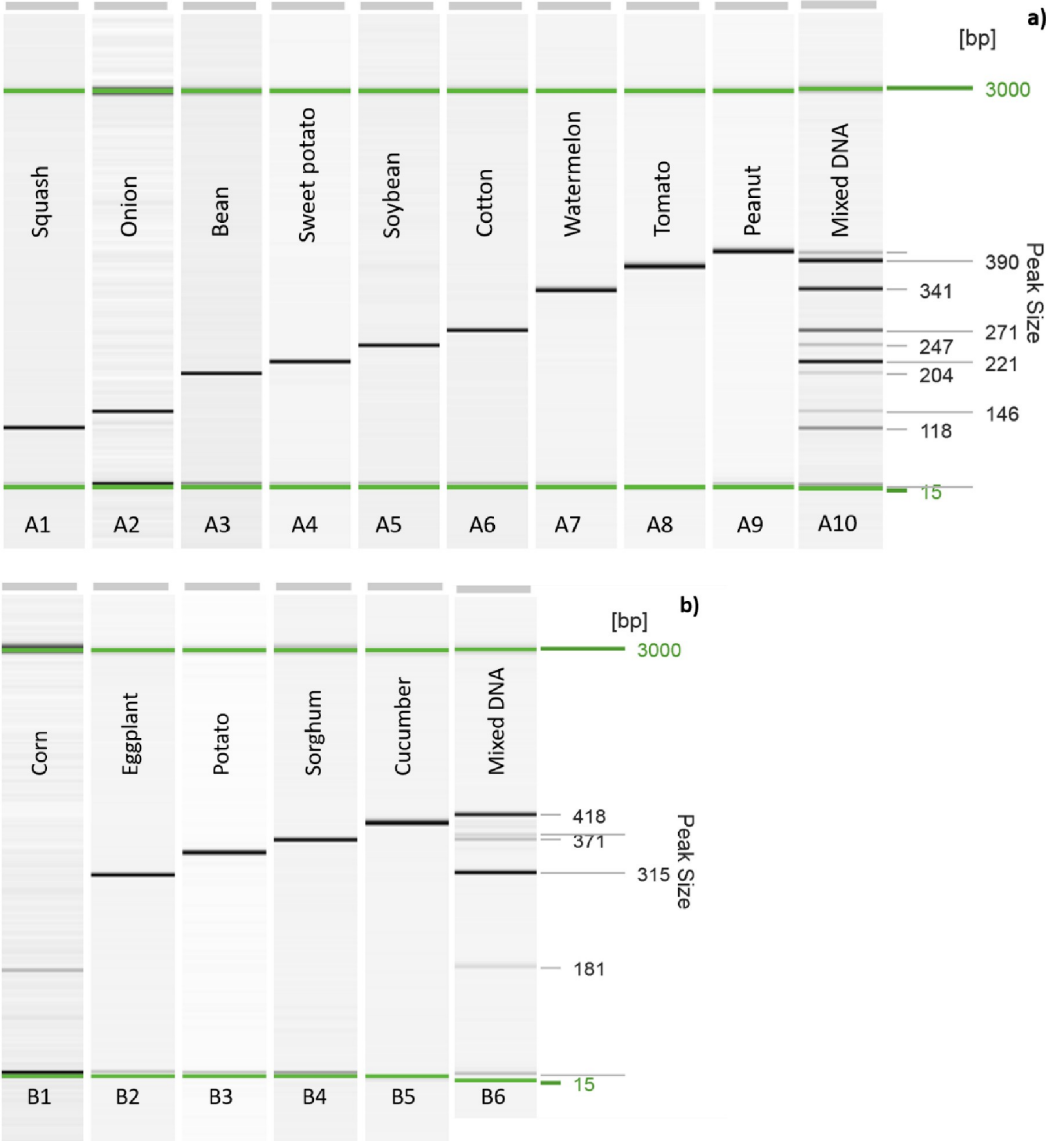
The amplification of the plant target DNA using two working multiplex primer mixes (primer mix 1 & 2, panel a & b) containing several species-specific primers and their associated amplicon sizes (bp). The PCR products were visualized on a QIAxcel Advanced system, and Qiagen alignment markers were employed to separate amplicon sizes ranging from 15bp to 3000bp. The plant species were ordered by expected base pair sizes, to indicate the expected ranges of band sizes using the designed primers. Panel a) consisted of the result of multiplex PCR on a single target species from left to the right A1-A9: *Cucurbita pepo*, *Allium cepa*, *Glycine max*, *Ipomoea batatas*, *Phaseolus vulgaris*, *Gossypium arboretum*, *Citrullus lanatus*, *Solanum lycopersicum* and *Arachis hypogaea*, respectively, followed by A10) their mixed target species DNA sample. Panel b) consisted of the result of multiplex PCR from left to right B1-B5 including *Zea mays*, *Solanum melongena*, *Solanum tuberosum*, *Sorghum bicolor* and *Cucumis sativus*, respectively, followed by B6) their mixed target species DNA sample.

The compatibility and sensitivity of primers were tested within various mixes to create two working multiplex PCR primer mixes (Table 2, Fig 1). Some primers were not compatible with others within the first mix, including sorghum and potato. Therefore, a second primer mix was required to effectively screen for all species. Furthermore, the expected band size for some of the primers was similar, resulting in a need to separate them in order to definitively conclude a plant species (e.g., *A*. *hypogaea* and *C*. *sativus*). Using primer sensitivity results as a guide, the PCR conditions were optimized testing different concentration of primers (0.1–0.5 μM) within the mixes. The final primer mixes resulted in the efficious detections as well as yielding PCR products with the highest and most equivalent concentrations of PCR products for each primer pair in the multiplex PCR (Tables 2 and 3).

### 3.3 Detectability half-life

**Fig 2:**
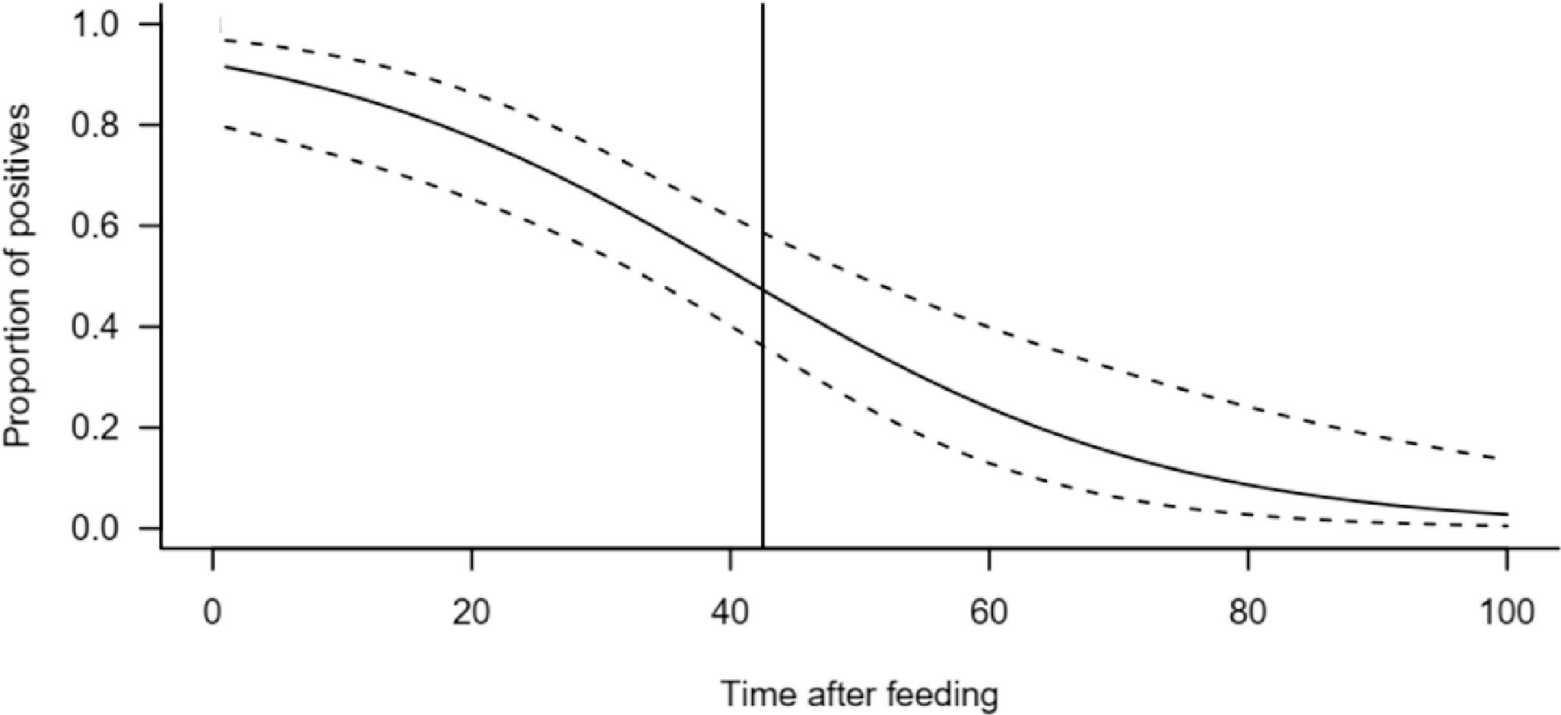
The proportion of *Melanoplus ponderosus* positives for cotton DNA at the intervals of 0, 12, 24, 36, 48, 72 and 96 h post-feeding, using the multiplex primer mix. The solid curve line represents the fitted probit model accompanied it’s of 95% confidence intervals (dotted lines). The solid vertical line is the time at which 50% of individuals are expected to test positive for plant DNA in their gut (half-life). R statistical software was used to create this figure.

**Fig 3:**
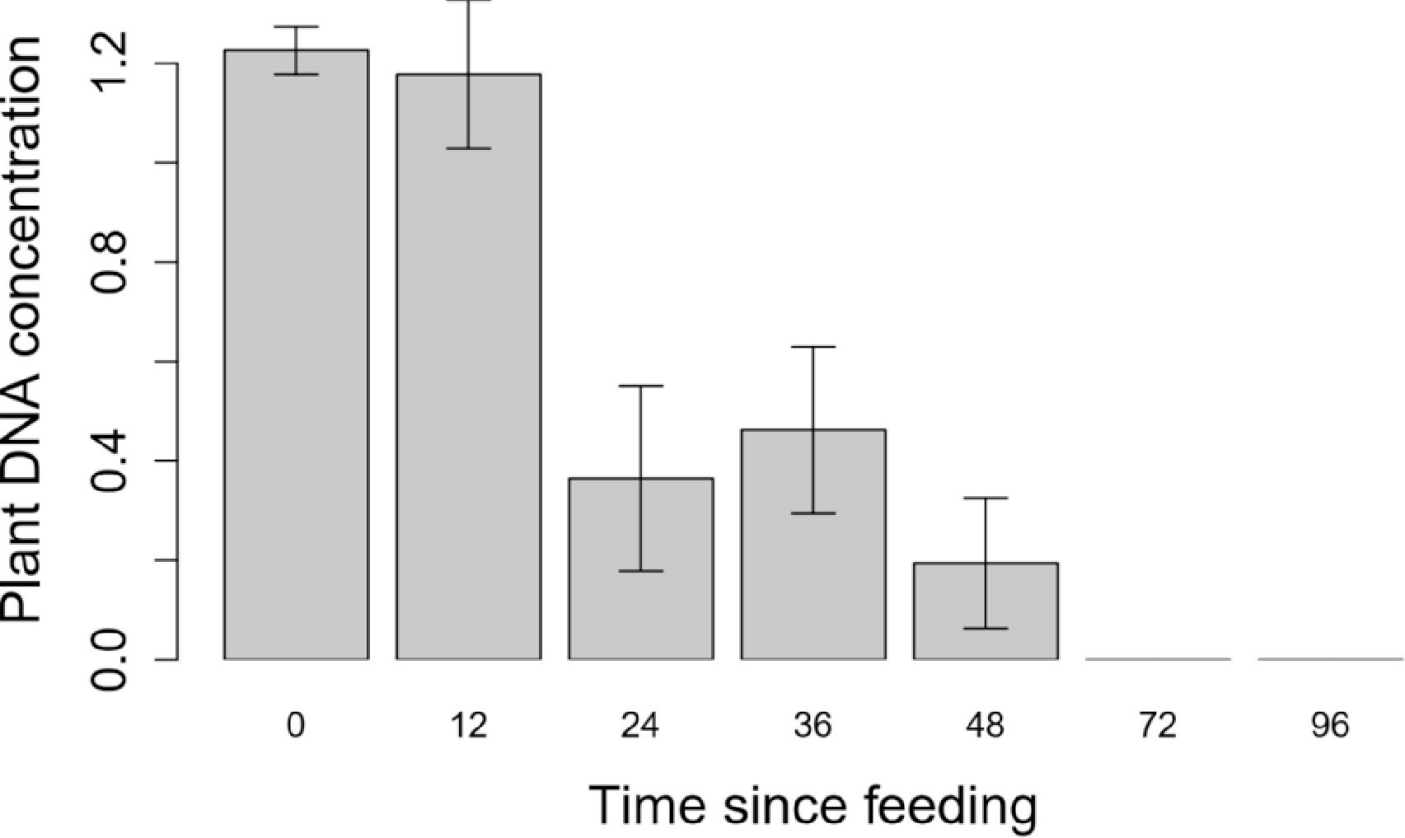
The concentration of remaining plant DNA ± SE in the gut of *Melanoplus ponderosus* after feeding at intervals of 0, 12, 24, 36, 48, 72- and 96-hours post-feeding, using the multiplex primer mix. R statistical software was used to create this figure.

The detectability half-life for cotton DNA in the gut of *M*. *ponderosus* was up to 42.5 h after feeding (Fig 2). The slope of the probit model significantly differed from zero (slope = -0.05999, z-value = -4.51, df = 68, P<0.0001). Similarly, the concentration of the remaining plant DNAs decreased over-time detected by the multiplex primer pair used, where the concentration of remaining cotton plant DNA in the gut of *M*. *ponderosus* dropped from 1.21 (ng/μL) at a 0 h, to lower than 0.22 (ng/μL) at 48 h (Fig 3).

### 3.4 Field-collected grasshopper screening

**Fig 4:**
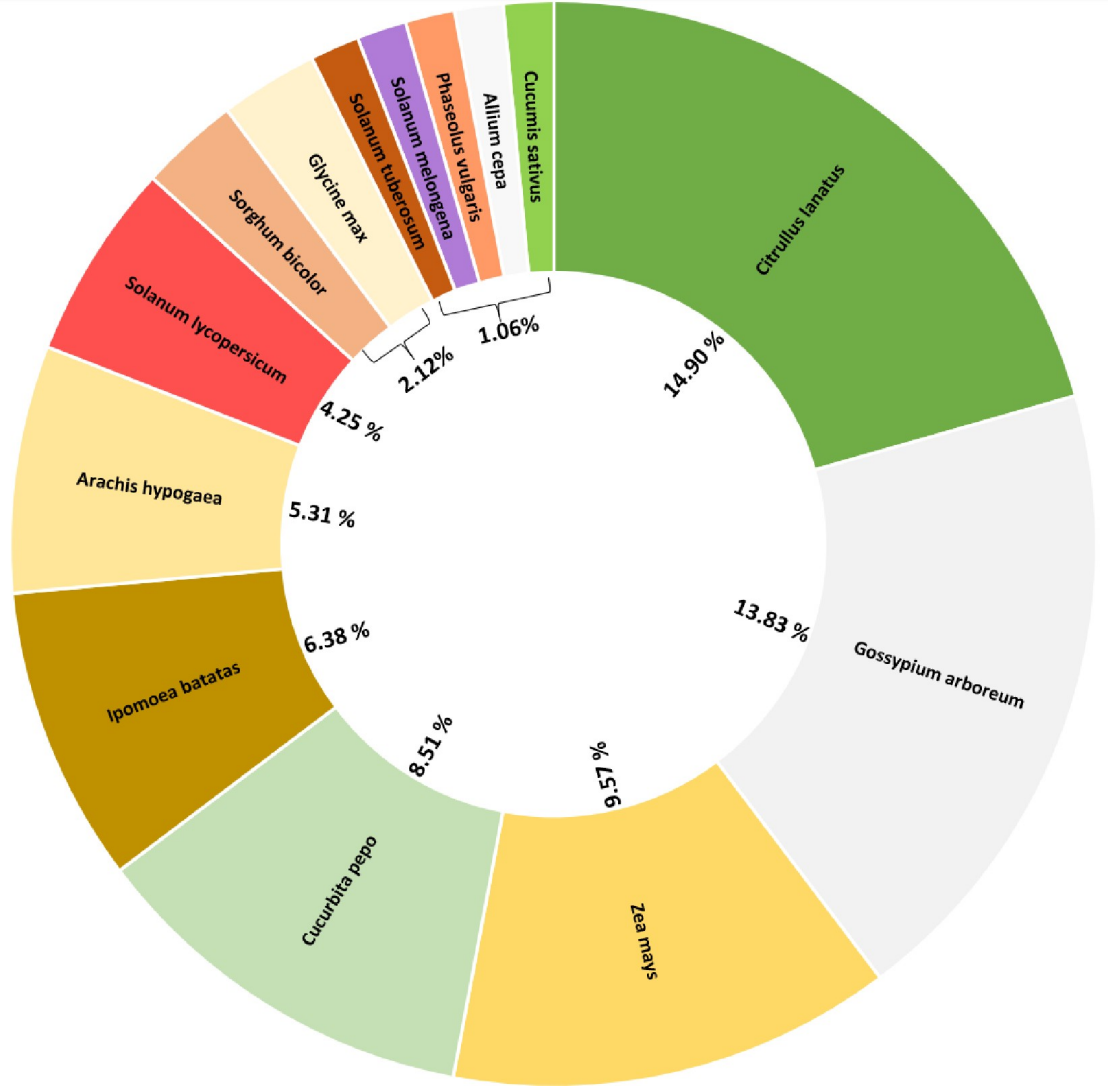
The frequency of feeding on 14 different crop plant species by *Melanoplus ponderosus* in South Georgia agricultural fields. The percentages of feeding on each plant species were calculated by dividing the total number of positives for each plant by the total number of tested individuals (94 individuals), multiplied by 100. The Microsoft PowerPoint program was used to create this figure.

Using these two primer mixes, 94 field-collected *M*. *ponderosus* were screened, out of which 47 individuals contained DNA from one or multiple crop targets in their gut content. One individual contained DNA from 5 target species, three individuals contained DNA from four target species, three individuals had DNA from three target species, six individuals had DNA from two target plant species, and thirty-five individuals only had one target species DNA in their gut. The feeding frequency on *C*. *lanatus* was higher, followed by *G*. *arboretum*, *Z*. *mays*, *C*. *pepo*, *I*. *batatas* and *I*. *batatas* with 14.90, 13.83, 9.57, 8.51 and 6.38%, respectively. The frequency of feeding was generally low or negligible for the other seven crop plant species with about 1% of individuals being positive for these crops (Fig 4).

## 4 Discussion

Our results show that the two working multiplex primer mixes designed in this study are specific to their target, and are sensitive to low concentrations of their target plant DNA in the gut content of polyphagous insect pests. Furthermore, our results show that primer pairs within each mix are compatible with one another, enabling the identification of 14 plant species within the guts content of insect pests using only two multiplex PCR reactions. We also report an optimized PCR assay condition that maximized the plant target detection within pest gut contents.

Several DNA regions have been used to date to develop specific and universal primers for the detection of animal and plant DNA in the gut of insect pests or predators [3]. Cytochrome c oxidase subunit 1 (CO1) is shown to be an excellent region for specific primer design for various insects, to screen for predator-prey interactions or estimate phylogenetic relationships [4, 64]. For plant identification, however, due to the lower rate of evolution in the CO1 region, the use of CO1 to differentiate plant species is inappropriate and impractical [65]. Hence, other DNA regions with a higher rate of evolution, such as rbcl, trnH-psbA, and ITS have been used to develop species-specific or universal plant primers for plant identification and polyphagous pest dietary evaluation [66, 67].

Given the difficulties of finding plant species-specific regions, most studies to date have used DNA metabarcoding approaches using universal rbcl and trnL primers to screen polyphagous pest diet in agroecosystems [20, 38–41, 65]. Few studies have attempted to design and report crop plant species-specific primers. Using the internal transcribed spacer (ITS) region, Pumarino et al. [33] reported a specific primer for tomato, which was used to screen omnivores for evidence of feeding on tomato plants. Similarly, Wang et al. [24] reported specific primers for cotton and mungbean from the trnL-trnF region of chloroplast DNA and used these primers to explore the movement of *Apolygus lucorum* (Heteroptera: Miridae) between cotton and mungbean fields. Currently, only one study [29], using chloroplast region (trnL-F cpDNA region) developed and reported several multiplex primer mixes for crop and non-crop plant dietary detection in insect pests, highlighting a need for additional studies. However, our study is the first to design and optimize a plant species-specific primer mix that can successfully detect 14 target crop plant DNA within two PCR reactions, which significantly eases plant molecular detection. Further, our primer mixes are the first to exclusively target economically important crops in agroecosystems, which could be used to screen various polyphagous pests (e.g., birds, mammals and insects) for evidence of feeding on these important crops in agroecosystems.

To our knowledge, no study has used full genome sequences to design crop plant species-specific primers for multiplex settings. While some earlier studies used chloroplast genome sequences for phylogenetic analysis and species identification [24, 42–44], we found that the chloroplast region is too conserved for designing plant-species specific primers for most of our target plant species. Therefore, for the first time, we have explored various regions of the plant genome and designed specific crop plant primers from VE-1 genes for *S*. *lycopersicum*, pathogenesis related protein-coding gene 2 (PR-2) in squash, PR-5 in *A*. *cepa*, and PR-4 region in all other plant species. Overall, our results suggest that designing primers based on sequences from whole-genome sequences, instead of chloroplast sequences, for plant species-specific primer design is an effective alternative approach that can significantly increase the possibility for plant-specific primer design for several plant species in agroecosystems. Our results warrant that future efforts use the full-genome approach to significantly boost the available specific primer pairs for additional plant molecular identification and dietary research in agroecosystems.

Over the years, many scientists attempt to use different approaches from restriction fragment length polymorphism (RFLP) to metabarcoding and sequencing for the identification of plant samples [31, 32, 68, 69]. However, there are some disadvantages to using these techniques. Most of the currently developed methods for the identification of plant species involve DNA sequencing [70, 71], which entails high costs. RFLP is another powerful approach to differentiate several plant species within a sample using restriction enzymes [31, 32]. The presence of a high number of species in samples can diminish RFLP usability due to the presence of similar base pairs amplicon sizes within the gut content, which result in overlapping bands and detections [29]. So, one of the advantages of our method is the efficiency of the technique in detecting and differentiating more than nine plant species simultaneously in one PCR reaction. However, one of the constraints of our technique is that the identification of different plant species would be based on the amplicon sizes. So, when designing specific primers, it is crucial to ensure that all amplicon sizes are varied with at least 10 bp in length. Also, care is advised when checking the primers against all sets of different non-target plants whose DNA might be present in the sample [72]. Overall, our developed primer mixes add a new path to the number of approaches that can be used to screen polyphagous pest diets in agroecosystems, which is specific, sensitive, rapid and cost-effective.

In this study, we designed primer mixes that successfully detected 14 plant species from the gut content of *M*. *ponderosus*, indicating a wide range of host crops this polyphagous pest consumes in the Georgia agricultural fields. The highest incidence of feeding observed was for watermelon, cotton and corn (Fig 4). Consistent with results from [47], our results confirmed that grasshoppers, such as *M*. *ponderosus*, feed on a wide range of crop plants in agroecosystems. Recent studies of grasshopper diets focus primarily on grasses and non-crop host plants [73]. Isley [47] study listed corn, cotton, sunflower, wheat, tobacco and sugarcane as potential host plants of grasshoppers in laboratory choice tests. Our results added several new host crop species such as watermelon, bean, eggplant, peanut to the diet of grasshoppers in agricultural fields. However, it needs to be pointed out that crop mixtures are frequently changing in the southeast and sub-tropical agroecosystems, and later in the season a polyphagous pest diet could certainly change depending on the type of crops available in agroecosystems. Our results further indicated that the designed primer mix can successfully be used to evaluate the diet of chewing insect pests within or between the cropping system. Hence, future studies should be conducted to test the efficacy of these primer sets for dietary detection of sucking pests. An earlier study by Wang et al. [24] reported the successful use of plant-specific primers for the detection of cotton and mungbean in the gut of *A*. *lucorum* (Heteroptera: Miridae), a sucking pest. This suggests that singleplex and multiplex PCR could be potentially used to screen sucking pest diets in agroecosystems, which warrants future studies.

Estimating the detectability half-life for prey/plant DNA in an insect gut enables standardization of the frequencies of molecular detections, and provides a window of detection for recent feeding [74]. While many studies estimated the detectability half-life for insect prey DNA in the gut of predators (reviewed by Greenstone et al., [74]), such estimation has seldom been assessed for plant DNA decay in herbivore guts [33, 34, 37]. We found that plant DNA remained detectible in grasshopper guts with a 50% probability for up to 40h post feeding, providing further support for the sensitivity of the designed primers for pest diet breadth evaluations. Pumarino et al. [33] found significant differences in the detectability half-life for tomato DNA in the gut of three insect predator and pest species, where it was shortest for the predatory bug, *Macrolophus pygmaeus* with 5.8h, followed by herbivory moths *Helicoverpa armigera* and *Tuta absoluta* with 27.7h and 28.7h, respectively. Further, using general plant primers, Staudacher et al. [36] found significant differences in the detectability half-life for six plant species in the gut of wireworm (Agriotes), suggesting that various plant DNA decay at a different pace in the gut of the same pest species. Altogether, these two studies suggest that the half-life should be estimated for each insect predator and pest species, and should further be estimated for a different type of diet DNA (e.g., plant or insect). Therefore, future studies using the multiplex PCR primer mixes should consider estimating detectability half-life for each pest and plant species under study, to ensure a standardized frequency of detection that would result in a justifiable conclusion regarding diet share of each plant species in polyphagous pest gut content.

The understanding of polyphagous pest diets and their natural enemies in agroecosystems is of major importance for designing habitat management strategies to minimize crop damage both directly through reduction of pest preferred host plants by rotation or indirectly by the provision of carbohydrate resources for natural enemies that boost their potential and result in higher pest control services [16, 75]. Insect pest and predator dietary information can further be used to develop push-pull strategies based on pest and predator dietary preferences to further reduce damage to the cash crop by diverting pests to the trap crop, and also attract more natural enemies into the cash crop system, as shown in previous studies [76, 77]. Several landscape-scale studies have been conducted to determine the pattern of insect movement between habitats in an agricultural landscape, using bi-directional malaise traps [78], molecular mark-recapture [25] and isotope techniques [27]. These authors investigated the movement of polyphagous insect pests and generalist predators to understand what habitats could provide them with alternative resources, and act as a reservoir from which pests and natural enemies spillover into agricultural fields. However, insect movement between habitats should not be the primary means of inferring their diet in agroecosystems. Detection does not reveal the true intention of insect pest or natural enemy for visiting a habitat. Using MGCA with specific plant and insect primers significantly boosts ecologists’ understanding of complex plant-pest-natural enemy interactions in agroecosystems. While the explanation for an insect pest visiting a crop field might be simple, for natural enemies it can be more complicated, as they are often omnivores and feed on both insect prey and plant carbohydrates [33]. Therefore, using MGCA can reveal the true purpose for predator movement to crop fields, by revealing their plant and insect diet in each habitat. Overall, the use of MGCA through specific plant primers designed in this study and Wallinger et al. [29], could have broad implications for insect pest management in agroecosystems and warrant future studies to fully explore and harness the knowledge acquired by employing MGCA to understand plant feeding and host use of common pests.

## 5 Conclusions

In this study, we report two optimized multiplex primer mixes for identifying recent feeding on plants, which could significantly ease the dietary evaluation of various polyphagous pests in agricultural environments. The designed multiplex PCR method provides rapid polyphagous pest dietary evaluation, with relatively low costs and time investment. Overall, this study provides a powerful tool to screen the diet of pests for 14 important crop plant species and exemplifies the broad dietary information a multiplex PCR approach can offer. These primer mixes also have implications in agroecological studies. Herbivore screening for recent feeding on plants can provide a cost-effective method for determining dietary breadth, tracking movement patterns between hosts in agricultural landscapes, and developing comprehensive host-herbivore networks to aid in designing habitat management practices that minimize benefits to pests.

## Supporting information

S1 File (ZIP)
